# Development of the Pulmonary Hypertension Functional Classification Self-Report: a patient version adapted from the World Health Organization Functional Classification measure

**DOI:** 10.1186/s12955-021-01782-0

**Published:** 2021-08-24

**Authors:** Kristin B. Highland, Rebecca Crawford, Peter Classi, Ross Morrison, Lynda Doward, Andrew C. Nelsen, Howard Castillo, Stephen C. Mathai, Hilary M. DuBrock

**Affiliations:** 1 Cleveland Clinic, Cleveland, OH USA; 2 RTI Health Solutions, The Pavilion, Towers Business Park, Wilmslow Road, Didsbury, Manchester, M20 2LS UK; 3 United Therapeutics, Durham, NC USA; 4 Johns Hopkins University School of Medicine, Baltimore, MD USA; 5 Mayo Clinic, Rochester, MN USA

**Keywords:** Pulmonary arterial hypertension, Pulmonary hypertension, WHO functional class, Qualitative, Patient-assessed, PH-FC-SR

## Abstract

**Background:**

Pulmonary arterial hypertension (PAH) is characterized by progressive limitations on physical activity, right heart failure, and premature death. The World Health Organization functional classification (WHO-FC) is a clinician-rated assessment used widely to assess PAH severity and functioning, but no equivalent patient-reported version of PAH symptoms and activity limitations exists. We developed a version of the WHO-FC for self-completion by patients: the Pulmonary Hypertension Functional Classification Self-Report (PH-FC-SR).

**Methods:**

Semistructured interviews were conducted with three health care providers (HCPs) via telephone to inform development of the draft PH-FC-SR. Two rounds of semi-structured interviews were conducted with 14 US patients with a self-reported PAH diagnosis via telephone/online to elicit concepts and iteratively refine the PH-FC-SR.

**Results:**

HCPs reported that the WHO-FC was a useful tool for evaluating patients’ PAH severity over time and for making treatment decisions but acknowledged that use of the measure is subjective. Patients in round 1 interviews (n = 6) reported PAH symptoms, including shortness of breath (n = 6), fatigue (n = 5), syncope (n = 5), chest pains (n = 3), and dizziness (n = 3). Round 1 patients identified challenges with the original WHO-FC, including comprehensibility of clinical terms and overlapping descriptions of class II and III, and preferred the Draft 1 PH-FC-SR over the original WHO-FC. After minor changes were made to Draft 2, round 2 interviews (n = 8) confirmed patients understood the PH-FC-SR class descriptions, interpreting them consistently.

**Conclusions:**

The HCP and patient interviews identified and confirmed certain limitations inherent within the clinician-rated WHO-FC, including subjective assessment and overlapping definitions for class II and III. The PH-FC-SR includes patient-appropriate language, symptoms, and physical activity impacts relevant to patients with PAH. Future research is recommended to validate the PH-FC-SR and explore its correlation with the physician-assessed WHO-FC and other outcomes.

## Background

Pulmonary arterial hypertension (PAH) is a rare, progressive, and debilitating subtype of pulmonary hypertension (PH) that commonly leads to right heart failure (HF) and death [1]. Three to five times more likely to occur in women than men, PAH usually affects women aged 30 to 60 years [2]. Symptoms of PAH include dyspnea, fatigue, weakness, angina, and syncope, all of which become more pronounced with worsening PAH severity [1]. As PAH progresses, patients often experience diminished health-related quality of life (HRQOL) and increasing functional limitations [1, 3]. Specifically, patients with PAH experience impaired general health and physical and social functioning and often are severely restricted in their physical activity and ability to work [1, 3, 4].

The World Health Organization functional classification (WHO-FC) is a tool used to measure disease severity in patients with PAH whereby health care providers (HCPs) use patient reports of symptom experience and activity limitations to make their assessment. The WHO-FC was adapted in 1998 for use with patients with PAH based on the New York Heart Association (NYHA) classification, a 4-point index of functional status of patients with HF [5, 6]. Patients assessed by the WHO-FC are allocated into class I, II, III, or IV; a higher class is indicative of more severe disease (i.e., greater functional impairment).

The WHO-FC is widely used in clinical settings to support treatment decisions and monitor disease progression and in PAH clinical trials as an enrollment criterion and key study outcome [1, 6–11]. The European Society of Cardiology’s Clinical Practice Guidelines recommend the WHO-FC to assess the severity of a patient’s PAH [12], and the measure is generally considered a significant prognostic indicator in PAH [13, 14]. It is used as a variable in a number of PAH risk calculators, including the Registry to Evaluate Early and Long-Term PAH Disease Management (REVEAL) risk calculator (REVEAL 2.0) and risk assessment tools based on results from the Comparative, Prospective Registry of Newly Initiated Therapies for Pulmonary Hypertension (COMPERA) and the Swedish PAH Register (SPAHR) [15–18]. The WHO-FC has also been shown to correlate with exercise capacity, hemodynamics, right heart function, N-terminal pro–brain natriuretic peptide (NT-proBNP)/BNP, and survival [19–21]. Importantly, for tracking patient progression, the WHO-FC also appears sensitive to therapeutic drug regimen changes [11, 22, 23].

The WHO-FC is an important part of the continuous assessment of patients with PAH. Having an available patient-completed version of the WHO-FC would provide greater versatility for its use, enabling the collection of functional class data in research settings where it is not feasible to collect clinician-completed data (e.g., in telehealth); allowing for longitudinal patient self-monitoring of disease progression in clinical trials or in clinical practice; and facilitating improved communication between patient and physician in regards to disease status. Thus, the objective of this qualitative interview study was to develop a version of the WHO-FC that can be self-completed by patients, without significant deviation from the original WHO-FC: the PH Functional Classification-Self-Report (PH-FC-SR).

## Methods

**Fig. 1:**
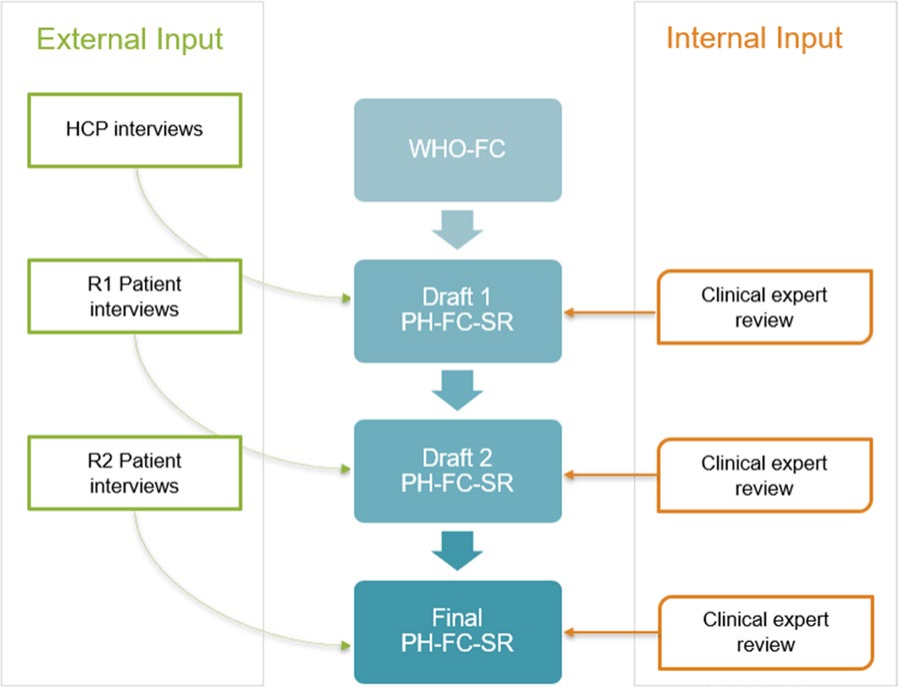
Pulmonary hypertension functional classification self-report development process

The development of the PH-FC-SR followed an iterative process that included telephone interviews with HCPs with clinical expertise in PAH as well as patients with PAH. Input from a clinical expert also guided development of the study materials (Fig. 1). All interviews followed a semi-structured interview guide and were conducted by two experienced qualitative researchers—one facilitator and one note-taker (LD, RC, and/or RM). The WHO-FC was adapted to a self-reported version with permission from Dr Stuart Rich, editor of the Executive Summary from the World Symposium on Primary Pulmonary Hypertension cosponsored by the World Health Organization [24].

This study was conducted in accordance with the amended Declaration of Helsinki. One of RTI International’s three institutional review boards approved the protocol and all study materials (STUDY00020846), and informed consent was obtained from all study participants.

### Health care provider interviews

To inform the development of the draft PH-FC-SR, initial qualitative interviews were conducted with three HCPs (KBH, SCM and HC), all of whom were PAH specialists based in the United States (US). These HCPs were identified based on their expertise and experience treating patients with PAH. The interviews were conducted via telephone and lasted up to 1 h. The interviews focused on how the HCPs currently use the WHO-FC in clinical practice; specifically, HCPs were asked open-ended questions about their understanding and interpretation of each WHO-FC class, their experiences with using the WHO-FC in clinical practice, and the process by which they use the WHO-FC to categorize patients with PAH.

Draft 1 of the PH-FC-SR was prepared using everyday (lay) language informed by the HCP interviews. It was designed to mirror the structure and content of the WHO-FC and was additionally informed by the self-assigned NYHA classification (SA-NYHA), a patient self-assessed version of the NYHA classification for HF [25]. The structure of the SA-NYHA was used to inform on the development of the first/initial draft PH-FC-SR.

The analysis of the HCP interviews used the interview field notes. The HCPs’ feedback on their interpretation and implementation of the WHO-FC in clinical practice was collated and summarized.

### Patient interviews

The draft PH-FC-SR was then refined and finalized iteratively across two rounds of qualitative interviews with patients. Patient participants were identified and recruited by a rare disease patient panel organization in the US. Eligible patients were aged 18 years or older with a self-reported diagnosis of PAH. Patients with any comorbid autoimmune connective tissue disease (e.g., scleroderma) were excluded from the round 1 interviews in order to limit noncardiopulmonary influences on Draft 1 of the PH-FC-SR. These types of comorbidities were not excluded from the round 2 interviews because of the study’s aim to increase the generalizability of the PH-FC-SR. Purposive sampling was used to ensure an inclusion of a range of WHO-FC classes. Individual interviews were conducted via a web-based platform (Zoom), lasted up to 1 h, and were audio recorded and transcribed. Fourteen unique patients with PAH participated; 6 in the first round of interviews, and 8 in the second round of interviews. These sample sizes were considered adequate for the assessment of content validity [26, 27].

The aim of the round 1 interviews was to refine the PH-FC-SR. The first round of patient interviews included a concept elicitation section designed to determine current symptoms and functional impact of PAH and to identify the language and terms use by patients to describe the impact of their PAH. This was followed by a cognitive debriefing of the original WHO-FC and Draft 1 of the PH-FC-SR. The round 1 interview guide included open-ended questions on symptoms, impact on daily life, and patients’ opinions on the newly developed PH-FC-SR. Findings from round 1 interviews provided preliminary evidence for the content validity of the new measure. Draft 2 of the PH-FC-SR was developed based on the round 1 interviews. The lead study clinician reviewed Draft 2 of the PH-FC-SR to ensure consistency with the WHO-FC prior to the round 2 interviews.

The second round of patient interviews focused on cognitively debriefing Draft 2 of the PH-FC-SR in order to determine the content validity of the new instrument. The final PH-FC-SR was developed following the round 2 interviews. The lead study clinician reviewed the final version of the PH-FC-SR to ensure consistency with the WHO-FC.

A qualitative description approach was used for the analysis of the patient interviews [28]. A coding framework was implemented to aid the analysis of the verbatim interview transcripts. The analysis was performed with the coding software ATLAS.ti (version 7.5; ATLAS.ti Scientific Software Development GmbH, Berlin, Germany).

## Results

### Participant characteristics

Two pulmonologists and one former PAH nurse practitioner with expertise in the evaluation and treatment of PAH for a combined duration of over 30 years were interviewed. One pulmonologist was primarily a clinician whose practice focused on PH; the other pulmonologist was a clinical researcher focusing on PH and outcome measures. The former PAH nurse practitioner worked with patients with PAH in the heart failure, advanced lung disease, and heart or lung transplant programs. All HCPs gained their experience in the US.

The demographic and self-reported PAH characteristics of the patient interviewees are presented in Table 1. The 14 interview participants had a mean (standard deviation) age of 46.0 (10.2) years, and 11 (78.6%) were female. Overall, 8 (57.1%) patients were working (either full time or part time), and the remaining 6 (42.9%) patients were on long-term sick leave or disability and/or were retired or semi-retired. Thirteen (92.9%) patients had some college education or higher.

**Table 1:** Patient interview sample characteristics

Characteristic	Round 1 (n = 6)	Round 2 (n = 8)	Total (n = 14)
Age, years			
Mean (SD), median	39.7 (3.7), 39	50.8 (11.1), 47	46.0 (10.2), 41.5
Range	35–46	40–68	35–68
Gender, n (%)			
Male	1 (16.7)	2 (25.0)	3 (21.4)
Female	5 (83.3)	6 (75.0)	11 (78.6)
Race, n (%)			
White/Caucasian	5 (83.3)	6 (75.0)	11 (78.6)
Black or African American	1(16.7)	1 (12.5)	2 (14.3)
Other	0 (0)	1 (12.5)^a^	1 (7.1)
Ethnicity, n (%)			
Not Hispanic or Latino	6 (100)	7 (87.5)	13 (92.9)
Hispanic or Latino	0 (0)	1 (12.5)	1 (7.1)
Relationship status, n (%)			
Married/living as married/civil partnership	2 (33.3)	4 (50.0)	6 (42.9)
Divorced/separated	0 (0)	1 (12.5)	1 (7.1)
Single	4 (66.7)	3 (37.5)	7 (50.0)
Employment status, n (%)^b^			
Working full time	2 (33.3)	2 (25.0)	4 (28.6)
Working part time	1 (16.7)	3 (37.5)	4 (28.6)
Long-term sick leave or disability	3 (50.0)	1 (12.5)	4 (28.6)
Retired/semi-retired	0 (0)	3 (37.5)	3 (21.4)
Highest level of education, n (%)^c^			
High school diploma or equivalent (e.g., GED)	1 (16.7)	0 (0)	1 (7.1)
Some college or associate’s degree	3 (50.0)	4 (50.0)	7 (50.0)
College graduate/bachelor’s degree	1 (16.7)	1 (12.5)	2 (14.3)
Advanced or professional degree	1 (16.7)	3 (37.5)	4 (28.6)
Health insurance, n (%)^d^			
Commercial insurance	3 (50.0)	3 (37.5)	6 (42.9)
Medicare, Medicaid, or public assistance program	3 (50.0)	6 (75.0)	9 (64.3)
General health, n (%)			
Excellent	0 (0)	0 (0)	0 (0)
Very good	0 (0)	2 (25.0)	2 (14.3)
Good	3 (50.0)	3 (37.5)	6 (42.9)
Fair	2 (33.3)	3 (37.5)	5 (35.7)
Poor	1 (16.7)	0 (0)	1 (7.1)
Affected by PAH, n (%)^e^			
A lot	3 (50.0)	4 (50.0)	7 (50.0)
Quite a lot	2 (33.3)	3 (37.5)	5 (35.7)
A little	1 (16.7)	1 (12.5)	2 (14.3)
Not at all	0 (0)	0 (0)	0 (0)
Other health conditions, n (%)			
Yes	2 (33.3)	5 (62.5)	7 (50.0)
No	4 (66.7)	3 (37.5)	7 (50.0)

GED = General Educational Development; PAH = pulmonary arterial hypertension; SD = standard deviation

^a^Patient reported race as white or Caucasian during screening

^b^Respondents could choose more than one category for employment status, which is reflected in the percentage

^c^All but 1 participant had some college education at the time of the interview; however, during screening, 2 patients reported “High school diploma or equivalent” as their highest level of educational achievement

^d^Respondents could choose more than one category for health insurance, which is reflected in the percentage

^e^The response scale for the impact of PAH was read out to the respondent as an ordered list

In round 1 interviews, 2 (33.3%) patients reported having other health conditions: 1 patient reported having sleep apnea and being overweight, and another patient reported having high blood pressure and being overweight. Among round 2 patients, 5 (62.5%) patients reported having other health conditions in addition to their PAH: 1 patient had congestive HF, scoliosis, anemia**,** and diabetes; one had congenital heart disease and high cholesterol; 1 had sleep apnea, was overweight, and had a high risk for recurring blood clots; and 1 had HIV and right HF. Across both rounds, 7 (50.0%) patients reported that their life was currently affected “a lot” by PAH.

### Development of the PH-FC-SR

#### Health care provider interviews

All HCPs found the WHO-FC to be a useful tool for evaluating patients’ PAH severity and functioning over time, making treatment decisions, and communicating patient disease severity among colleagues. Health care provider assessments of PAH were predominantly based on the patients’ responses to open-ended questions about their physical functioning and impact of symptoms. Although HCPs were aware of their patients’ clinical assessments (e.g., 6-min walk distance test), none of those interviewed stated that they considered this information in their formal assessment; instead, their assessment of WHO-FC class was based on their subjective assessment of patients during their clinical interview.

**Fig. 2:**
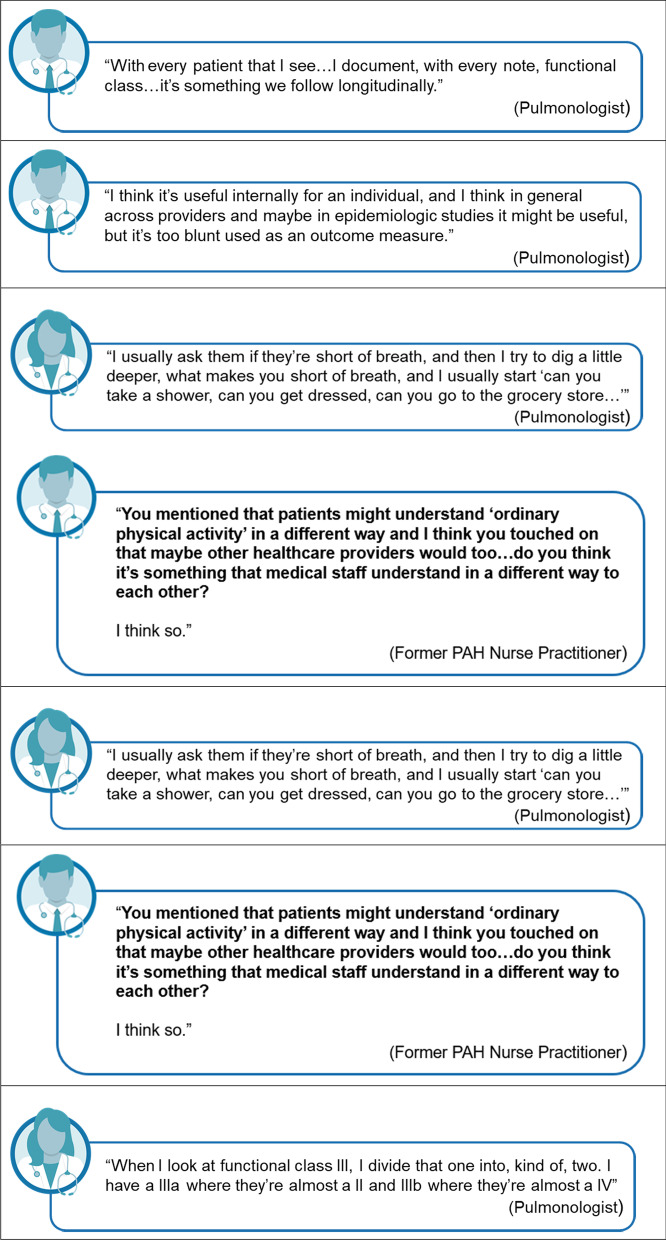
Health care provider understanding and usage of the WHO-FC

Taken together, the HCP interviews highlighted the subjective nature of WHO-FC implementation. Each HCP used their own interpretation of the classes when categorizing patients. The HCPs also noted that it could be challenging to distinguish between classes II and III, which led to the unofficial use of informal sublevels, particularly for class III (i.e., IIIa or IIIb). Figure 2 presents selected verbatim descriptions of how the HCPs understood the WHO-FC and used it in clinical practice.

Draft 1 of the PH-FC-SR was developed based on the results from the HCP interviews (Table 2). Brief instructions were added, clinical terms (e.g., “syncope” and “dyspnea”) were replaced with lay terms (e.g., “fainting” and “shortness of breath”), and examples of physical activities were added to illustrate the intended meaning of “ordinary physical activity” and “less than ordinary physical activity.”

**Table 2:** Pulmonary hypertension functional classification self-report class description tracking matrix

WHO-FC	Draft 1 PH-FC-SR	Draft 2 PH-FC-SR	Final PH-FC-SR
*Instructions*			
None included	Please select the description that best describes yourself:	Please select the description that best describes yourself:	Please place an X in the box next to the Functional Class that best describes yourself:
*Class I*			
Patients with pulmonary hypertension but without resulting limitation of physical activity. Ordinary physical activity does not cause undue dyspnea or fatigue, chest pain or near syncope	I have pulmonary hypertension, but my physical activity is not limited. I can do ordinary physical activity (e.g., household tasks, go to work, exercise) without getting short of breath, feeling tired, experiencing chest pains, or feeling like I may faint	I have pulmonary hypertension, but my physical activity is not limited I can do my regular day-to-day physical activity (e.g., household tasks, go to work, go to the store) and my usual exercise without getting short of breath or feeling tired or experiencing chest pains or feeling like I may faint	I have pulmonary hypertension, but my physical activity is not limited I can do my day-to-day physical activity (e.g., household tasks, go to work, go to the store) and my usual exercise without getting short of breath or feeling tired or experiencing chest pains or feeling like I may faint
*Class II*			
Patients with pulmonary hypertension resulting in a slight limitation of physical activity. They are comfortable at rest. Ordinary physical activity causes undue dyspnea or fatigue, chest pain, or near syncope	I have pulmonary hypertension and my physical activity is slightly limited. I feel comfortable at rest, but I experience shortness of breath, tiredness, chest pains, or feel like I may faint when I do ordinary physical activity (e.g., household tasks, go to work, exercise)	I have pulmonary hypertension and my physical activity is slightly limited I feel comfortable at rest. I can do my regular day-to-day physical activity (e.g., household tasks, go to work, go to the store) but it makes me feel short of breath or tired, or have chest pains or feel like I may faint	I have pulmonary hypertension and my physical activity is slightly limited I feel comfortable at rest. I can do my day-to-day physical activity (e.g., household tasks, go to work, go to the store) but it makes me feel short of breath or tired or have chest pains or feel like I may faint
*Class III*			
Patients with pulmonary hypertension resulting in a marked limitation of physical activity. They are comfortable at rest. Less than ordinary activity causes undue dyspnea or fatigue, chest pain or near syncope	I have pulmonary hypertension and my physical activity is noticeably limited I feel comfortable at rest, but I experience shortness of breath, tiredness, chest pains, or feel like I may faint when I do less than ordinary physical activity (e.g., washing, dressing, walking about the home)	I have pulmonary hypertension and my physical activity is noticeably limited I feel comfortable at rest. I can do the type of physical activity I have to do on a day-to-day basis (e.g., bathing, dressing, walking about the home), but it makes me feel short of breath or tired, or have chest pains or feel like I may faint	I have pulmonary hypertension and my physical activity is noticeably limited I feel comfortable at rest. I can do the type of physical activity I have to do on a day-to-day basis (e.g., bathing, dressing, preparing meals) but it makes me feel short of breath or tired or have chest pains or feel like I may faint
*Class IV*			
Patients with pulmonary hypertension with inability to carry out any physical activity without symptoms. These patients manifest signs of right heart failure. Dyspnea and/or fatigue may even be present at rest. Discomfort is increased by any physical activity	I have pulmonary hypertension and I am unable to carry out any physical activity without getting short of breath, feeling tired, experiencing chest pains, or nearly fainting. I sometimes get short of breath or tired when resting. I experience swollen ankles and a swollen stomach. I experience increasing amounts of discomfort with any physical activity	I have pulmonary hypertension and almost any physical activity makes me feel short of breath or tired, or have chest pains or nearly faint. I sometimes experience swollen ankles and/or a bloated stomach. I sometimes get short of breath or tired when resting. I experience increasing amounts of discomfort with any physical activity	I have pulmonary hypertension and almost any physical activity makes me feel short of breath or tired or have chest pains or nearly faint. I frequently experience swollen ankles. I may have a bloated stomach. I may get short of breath or tired even when resting. I experience increasing amounts of discomfort with any physical activity

PH = pulmonary hypertension; PH-FC-SR = PH Functional Classification Self-Report; WHO-FC = World Health Organization functional classification

#### Patient interviews

**Fig. 3:**
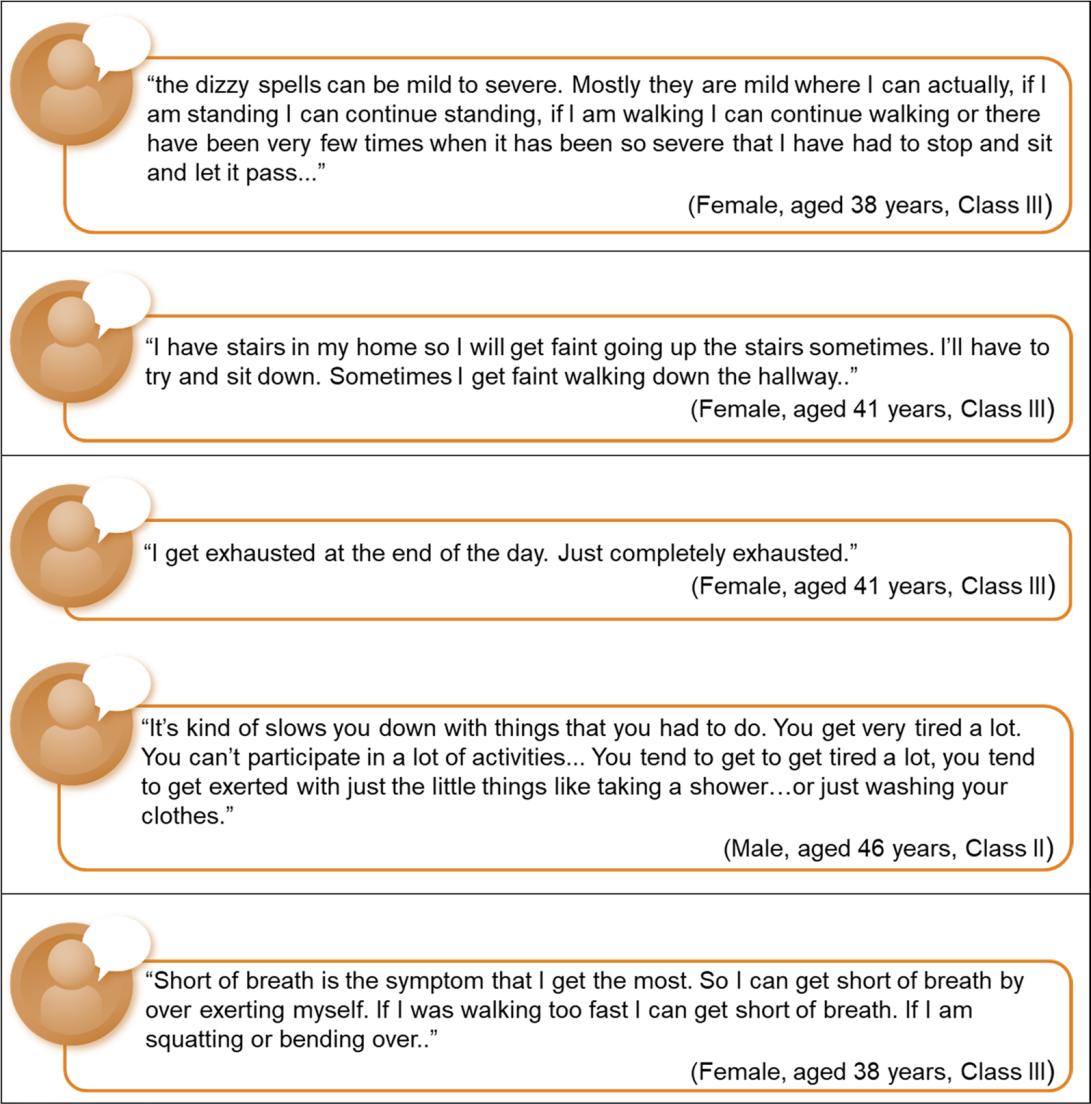
Patient-reported symptoms and impacts of pulmonary arterial hypertension

During the concept elicitation portion of the round 1 interviews, patients reported experiencing PAH symptoms, including shortness of breath (100.0%, n = 6), fatigue or tiredness (83.3%, n = 5), syncope or near syncope (83.3%, n = 5), chest pains (50.0%, n = 3), and dizziness (50.0%, n = 3) (Table 3). Most patients described having to alter their daily routines and activities to accommodate their PAH (e.g., leaving enough time to complete tasks, getting help with household tasks [grocery shopping, cleaning, etc.], and ensuring the accessibility of buildings). Figure 3 presents selected verbatim descriptions of the symptoms and impacts of PAH that patients experienced.

**Table 3:** Pulmonary arterial hypertension symptoms: round 1 patients

PAH symptom	Frequency (n = 6)	Included in WHO-FC
Shortness of breath	6	Yes
Fatigue or tiredness	5	Yes
Syncope or near syncope	5	Yes
Chest pains	3	Yes
Dizziness	3	No
Swelling	2	Yes
Purple lips	1	No
Reduced muscle strength	1	No
Brain fog	1	No
Heart palpitations	1	No

PAH = pulmonary arterial hypertension; WHO-FC = World Health Organization functional classification

Patients interviewed in round 1 had difficulty comprehending the clinical terms in the WHO-FC, and 3 (50.0%) patients suggested that lay terminology should be used. The class I and class IV descriptions were well understood by all patients. Some patients found class II and class III more difficult to define, with 4 (66.7%) patients suggesting that there was overlap between class II and class III.

Draft 1 of the PH-FC-SR was described as relatable and easy to understand (Table 4). All patients interpreted each description as intended, and all preferred Draft 1 of the PH-FC-SR to the original WHO-FC. Draft 2 of the PH-FC-SR was developed based on the results of the round 1 interviews and reviewed by the lead study clinician (see third column of Table 2). The sentence structure was amended to match that of the original WHO-FC: “Ordinary physical activity” was replaced with “day-to-day physical activity” in class I and class II; “Exercise” was removed as an example of day-to-day physical activity and replaced with “going to the store” but included in the class I description and rephrased as “my usual activities” and “less than ordinary physical activity” was replaced with “the type of physical activity I have to do on a daily basis.”

**Table 4:** Patient feedback on the draft pulmonary hypertension functional classification-self-report

Topic	Round 1 Quotations	Round 2 Quotations
General comments	“*It fits a lot of my symptoms and it also describes some of the things that I go through.*” Male, 46 years, Class II	“*Well, I think it’s pretty clear and concise.”* Female, 59 years, Class III *“Well I like how its worded and it sounds a bit more personal than the other WHO categories.”* Female, 40 years, Class III
Instructions	“**…*so do you think these instructions are clear and easy to understand?*** *Yes* ***And is it clear what they are asking you to do?*** *Yes*.” Female, 41 years, Class III	***“Did you find those instructions helpful or useful?”*** *“Yes.”* ***“Is there anything that we could kind of do to make them more easy to understand?”*** *“No, not that I can think of off the top of my head.”* Male, 42 years, Class II
Description I	“*I think the question people are going to go is, what is ordinary physical activity but when you give them choices like that, they’re like 'oh okay, they are talking about this, this and this.' So, I think that’s fairly clear and I can’t think of anything to add.*” Female, 38 years, Class I*	*“This means to me like normal people. I mean, they can do whatever they want to do, they can go to work, they can go to school, you know, you’re not getting out of breath, you’re not having any chest pains. To me, this is what a normal person’s life would be like.”* Female, 63 years, Class III
Description II	“…*it’s easy to understand.*” Female, 35 years, Class III	“*I would say that they have a very light effect of PAH symptoms but not significant enough to really slow them down with anything that they do, but just a slight sensation of shortness of breath or just occasional feeling like they’re faint.*” Female, 59 years, Class III
Description III	“*So it’s a noticeable limitation with my physical activity and what I like about this is that it not only says less than ordinary physical activity but it goes into what less than ordinary is. So showering and dressing, when somebody in a Class II they might not have any limitations but someone in a Class III might be symptomatic when they are taking a shower or dressing. So you know it gives you examples of what’s considered less than ordinary.”* Female, 38 years, Class III	“*Unlike the Class II where you just have an ever so slight impact, that only you personally feel, where the noticeably limited means not just the way you feel, but other people can see that you’re laboring as well.”* Female, 59 years, Class III
Description IV	“*It means that I am pretty much symptomatic with all physical activity and that may even include at rest.*” Female, 38 years, Class III	“*You are having lots of symptoms; you are having a lot of heart issues and the swollen ankles and bloated stomach. Sleeping is almost impossible, and you are constantly short of breath and exercise will be, well any kind of physical activity will be severely limited.*” Male, 42 years, Class II

PAH = pulmonary arterial hypertension; WHO = World Health Organization

Draft 2 of the PH-FC-SR was described as clear and easy to use (Table 4); the class descriptions were well understood and interpreted consistently across patients. Almost all patients found it easy to choose a functional class; only 1 (16.7%) patient found it difficult to choose a functional class because of a perceived overlap between the class II and class III descriptions. The patient-suggested changes to Draft 2 of the PH-FC-SR were generally idiosyncratic; no changes were suggested that had consensus among patients.

Patients in both interview rounds reported that they were familiar with the WHO-FC, but they did not discuss their WHO-FC with their HCP on a regular basis. In general, patients agreed with the clinician reported WHO-FC.

**Fig. 4:**
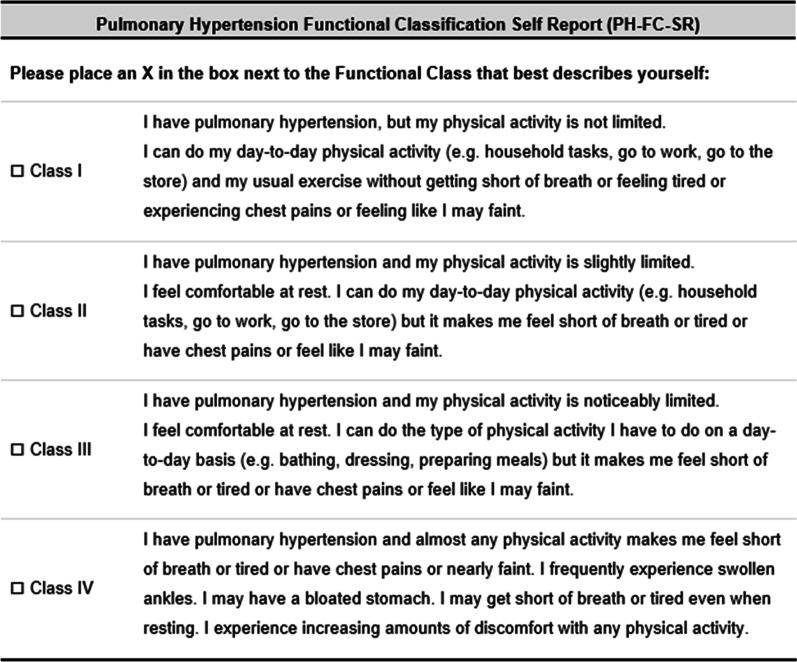
Final pulmonary hypertension functional classification self-report. Adapted from the Pulmonary Hypertension WHO Functional Classification System with permission from Dr. Stuart Rich, editor of the Executive Summary from the World Symposium on Primary Pulmonary Hypertension cosponsored by the World Health Organization [24]. The PH-FC-SR is freely available for use in clinical trials, clinical studies or other research settings. Users may not change, modify or add to the PH-FC-SR items and response options. Users may adapt or add to the PH-FC-SR instructions to suit the research study or the mode of questionnaire administration. The PH-FC-SR was developed and tested in United States (US)-English for use in the US. Users may freely prepare new language versions of the PH-FC-SR without permission from the authors. However, the process for translation must use the US-English version as the source language document, and the process for translation should conform to industry standards as set out in the ISPOR Good Research Practice Guidelines [40]. All studies should reference the US language development publication. This image is not covered by the article CC BY +CC0 license. This image is published under Creative Commons license CC BY ND: https://creativecommons.org/licenses/by-nd/2.0/. Image credit to United Therapeutics Corp. All reproductions must indicate “© 2021 United Therapeutics Corp., adapted with permission from Pulmonary Hypertension WHO Functional Classification System,” and all adaptions, including translations, must indicate “Adapted from PH-FC-SR © 2021 United Therapeutics Corp., which was adapted with permission from Pulmonary Hypertension WHO Functional Classification System.”

The final PH-FC-SR was developed based on the results of the round 2 interviews and reviewed by the lead study clinician (see Table 2). The instruction “Please select” was replaced with “Please place an X in the box.” Minor wording changes were made to aid readability: the example “walking about the home” in class III was replaced with “preparing meals,” “I sometimes experience swollen ankles and/or a bloated stomach” in class IV was replaced with “I frequently experience swollen ankles. I may have a bloated stomach,” and “I sometimes get short of breath or tired when resting” in class IV was replaced with “I may get short of breath or tired even when resting.” Figure 4 presents the final PH-FC-SR.

## Discussion

The PH-FC-SR has been adapted from the WHO-FC as a patient-completed assessment of functional class for individuals with PAH. Qualitative input from HCPs and patients indicates that the measure uses clinically relevant yet patient-friendly language and includes symptoms and physical activity impacts that are relevant to patients with PAH. Even if patients did not experience a specific symptom (e.g., fainting), they understood that this symptom could be relevant to other patients with PAH. Similarly, patients reported that the descriptions of physical activity limitations were consistent with their experiences of the impact of PAH.

Patient perceptions of symptoms and activity limitations are critical to characterizing the full impact of PAH, complementing clinicians’ subjective ratings of a patient’s symptoms, and other objective measures of disease severity. The importance of the patient perspective in the management of PH has been established [29] and is advocated by the FDA in their drive for patient-focused drug development [30]. In addition, HRQOL, as assessed by patient-reported outcome measures, is predictive of survival [31, 32] and clinical deterioration [33] in PAH. The PH-FC-SR provides a structured measure of patient-reported symptoms and activity limitations in PAH for use in a variety of settings, including routine clinical practice, research contexts (such as clinical trials), and patients' self-monitoring of their PAH at home. The PH-FC-SR enables comparison with physician assessments of functional class via the WHO-FC, as well as a means for patients to track their PAH severity longitudinally. More broadly, it transitions the WHO-FC from a clinician assessment into a patient-reported assessment.

Although this study represents the first initiative to adapt the WHO-FC measure for self-completion by patients, prior studies have aimed to adapt functional class assessments used in clinical practice—most notably, the NYHA classification index for individuals with HF. Kubo and colleagues [5] developed a questionnaire designed to determine NYHA class for use in nonblinded multicenter trials. The questions, designed to “mimic a conversation between a physician and patient,” were administered to patients by clinic staff; answers were scored using a scoring tool and converted into a NYHA functional class. The resulting questionnaire achieved approximately 60% concordance with the NYHA class assigned by treating physicians. Holland et al. [25] later adapted the NYHA classification to create a patient-reported version, the SA-NYHA, to predict treatment outcomes in an observational analysis within a clinical trial. The study found that the SA-NYHA was predictive of increased hospitalization rates, worse HRQOL, and decreased survival. These studies demonstrate that established measures of functional class can be successfully adapted for self-completion by patients or for use in settings other than routine clinical practice.

Some limitations of this study should be considered, including the self-selecting nature of the recruitment process and the patient self-reported diagnosis and disease severity via the WHO-FC during screening. Another minor limitation of this study was that a majority of the patient sample identified as predominantly white or Caucasian (79%); however, the comparative distributions in other PAH studies provide some support that our patient sample was generally reflective of the distribution of PAH patients in terms of race and ethnicity [34, 35]. Moreover, the objective of this study was to produce a version of the WHO-FC that could be completed by patients; thus, patients from a range of educational levels were included in the interview sample to ensure the language register would be suitable for all patients. The mean age of the sample, 46.0 years, is potentially younger than that of the broader PAH patient population, although there is evidence to suggest that age varies by region [36, 37].

It also should be noted that the PH-FC-SR is a subjective, not an objective, assessment of PAH severity. Patients adapt to chronic conditions and as such may not realize the seriousness of their symptoms, which may potentially lead to an underestimation of PAH severity. Furthermore, although the PH-FC-SR has been tested only with PAH patients so far, the ultimate intention is for the PH-FC-SR to be suitable for use by all patients with PH. Thus, further work is required to confirm the appropriateness of the new PH-FC-SR in other PH populations.

## Conclusions

Prior research has shown that HCPs and patients with chronic heart or lung disease perceive disease severity differently [38]. Qualitative findings from this study further emphasize differences in how HCPs and patients perceive and classify the symptoms of PAH and its impact. Patients’ reports regarding stability of their PAH and class-specific symptoms were not always congruent with the HCP perspective. For example, although the WHO-FC only includes manifest signs of right HF—described by the HCPs as swollen ankles and a bloated stomach—in class IV patients, patients described experiencing these symptoms in all classes except class I.

Findings from this study corroborate several known challenges associated with the implementation of the WHO-FC, including subjective assessment, overlap between class descriptions, and interobserver variability [1, 8, 14, 39]. The PH-FC-SR provides utility for collecting functional class reports directly from patients in settings where it may not be feasible or practical to collect clinician-rated functional class. An additional benefit of the PH-FC-SR is that it negates the bias that may be introduced with clinician assessments in which clinicians may unknowingly account for other clinical tests that have been conducted and read prior to the assessment.

Results of this study provide supportive evidence of limitations of the WHO-FC and considerations for future research to improve the measurement of functional class from the patient perspective. Future studies are planned to evaluate the psychometric properties of the PH-FC-SR with focus on its agreement with a provider-assessed WHO-FC and general construct validity as assessed by its relationships with other outcomes in PAH, including HRQOL (e.g., 36-Item Short Form Health Survey [SF-36], EQ-5D, Pulmonary Arterial Hypertension–Symptoms and Impact [PAH-SYMPACT]). Further, the lack of concordance in interpretations of PAH between HCPs and patients identified in this study suggests the need for the development of a new patient functional classification tool that is predictive of mortality risk but goes beyond a lay translation of the original WHO-FC.

## Data Availability

To protect the confidentiality of the study participants, the interview data generated and analyzed during the current study cannot be shared.

## Abbreviations

BNP: Brain natriuretic peptide
FDA: Food and Drug Administration
GED: General Educational Development
HCP: Health care provider
HF: Heart failure
HRQOL: Health-related quality of life
NYHA: New York Health Association
PAH: Pulmonary arterial hypertension
PAH-SYMPACT: Pulmonary Arterial Hypertension–Symptoms and Impact
PH: Pulmonary hypertension
PH-FC-SR: Pulmonary Hypertension Functional Classification Self-Report
R1: Round 1
R2: Round 2
SA-NYHA: Self-assigned New York Health Association
SD: Standard deviation
SF-36: 36-Item Short Form Health Survey
SPAHR: Swedish PAH Register
US: United States
WHO-FC: World Health Organization Functional Classification
